# Foveal eversion patterns in diabetic macular edema

**DOI:** 10.1038/s41598-022-17555-8

**Published:** 2022-07-30

**Authors:** Alessandro Arrigo, Andrea Saladino, Emanuela Aragona, Alessia Amato, Luigi Capone, Lorenzo Bianco, Rosangela Lattanzio, Francesco Bandello, Maurizio Battaglia Parodi

**Affiliations:** Department of Ophthalmology, IRCCS San Raffaele Scientific Institute, via Olgettina 60, 20132 Milan, Italy

**Keywords:** Anatomy, Biomarkers, Diseases, Medical research

## Abstract

The aim of the present study was to describe foveal eversion patterns in diabetic macular edema (DME) and to assess their relationship with the course of the disease and the outcome. The study was designed as prospective, observational, with two years of follow-up. DME patients were divided in two groups, one treated by combined anti-VEGF injections and dexamethasone (DEX) implants, and the other treated by fluocinolone acetonide (FAc) implant with additional anti-VEGF retreatments if needed. Main outcome measures were foveal eversion prevalence, foveal eversion patterns, best-corrected visual acuity (BCVA), central macular thickness (CMT), structural OCT metrics, number of intravitreal injections. One hundred and forty-six eyes (146 patients; 80 males; mean age 67 ± 8 years) affected by already treated DME, with 84 eyes treated with anti-VEGF/DEX treatments (mean of 10 ± 3 injections) and 62 treated with FAc implant. Looking at the treatments administered before the inclusion into the study, 84 eyes (58%) were treated with anti-VEGF injections, whereas 62 eyes (42%) underwent a combination of anti-VEGF and corticosteroids implants. DME eyes showed statistically significant improvements of LogMAR BCVA and CMT over the 2-year follow-up. Foveal eversion was found in 83 eyes (57%), categorized as follows: Pattern 1a (16;19%); Pattern 1b (22;27%) and Pattern 2 (45;54%). BCVA improvement was detected in all the subgroups, excepting for Pattern 2, which showed also significantly worse structural OCT parameters. Pattern 1b and Pattern 2 were characterized by significantly higher prevalence of persistent DME (64% and 89% of cases, respectively). Foveal eversion patterns were correlated with progressively worse DME outcome. Foveal eversion may be associated to the loss of foveal homeostasis, with consequent poor response to intravitreal treatments and worse DME outcome.

## Introduction

Diabetic macular edema (DME) is a frequent complication of diabetic retinopathy (DR), leading to a decline in visual acuity^1,2^. In most cases, DME can be managed by means of intravitreal anti-VEGF and corticosteroids. Nevertheless, the evolution of DME is often unpredictable and may include remission, recurrence, and persistence^3–10^. Although investigated in previous studies, the factors leading to bad outcomes in DME are still poorly understood^11–15^. In a recent study, we highlighted the role of a new structural optical coherence tomography (OCT) biomarker, namely foveal eversion, in the diagnostic workup of DME patients^16^. Foveal eversion, which describes a central fovea with a completely convex profile, was associated with a significantly higher prevalence of persistent DME, than the normal foveal profile^16,17^, regardless of the administration of intravitreal anti-VEGF or corticosteroids drugs.

In the present study, we assessed the presence of further patterns of foveal eversion, resulting in different morphological and functional DME outcomes secondary to the intravitreal treatments.

## Materials and methods

The study was designed as observational prospective case series. Patients affected by DME were progressively recruited at the Department of Ophthalmology, San Raffaele Scientific Institute, Milan, Italy from January 2017 to January 2019. The study was approved by the Ethical Committee of San Raffaele Scientific Institute, Milan, Italy, and conducted in accordance with the Declaration of Helsinki. All patients gave signed informed consent before being included in the study.

The inclusion criterion was the presence in pseudophakic eyes of clinically significant DME, as detected by structural OCT, treated for at least six months before our baseline examination by means of intravitreal injections. Exclusion criteria were: naïve DME, phakic eye, other media opacities, uncontrolled glaucoma, any ophthalmic surgery in the 6 months prior to treatment, macular edema secondary to causes other than DME, and any ophthalmic or systemic disease potentially affecting the results of the study, follow-up interval major than 4 months within the entire observational study window. A two-year follow-up was planned for all the eyes included.

All the patients underwent complete ophthalmologic examination, including best corrected visual acuity (BCVA) evaluated by standard ETDRS charts, anterior and posterior segment slit lamp evaluation, and Goldmann applanation tonometry. Structural OCT examination (Spectralis HRA, Heidelberg Engineering; Heidelberg, Germany) was performed at each follow-up visit, with radial, raster and dense scans with a high number of frames (ART > 25), and enhanced depth imaging (EDI) to highlight choroidal structures. Follow-up examinations were planned according to ophthalmologists’ discretion and treatment strategy.

The quantitative analysis included the following parameters: central macular thickness (CMT), inner retinal thickness (IRT), outer retinal thickness (ORT), disorganization of the retinal inner layers (DRIL), epiretinal membrane (ERM), ellipsoid zone (EZ) and external limiting membrane (ELM) status, retinal hyperreflective foci (HF), and subretinal fluid (SRF).

**Figure 1:**
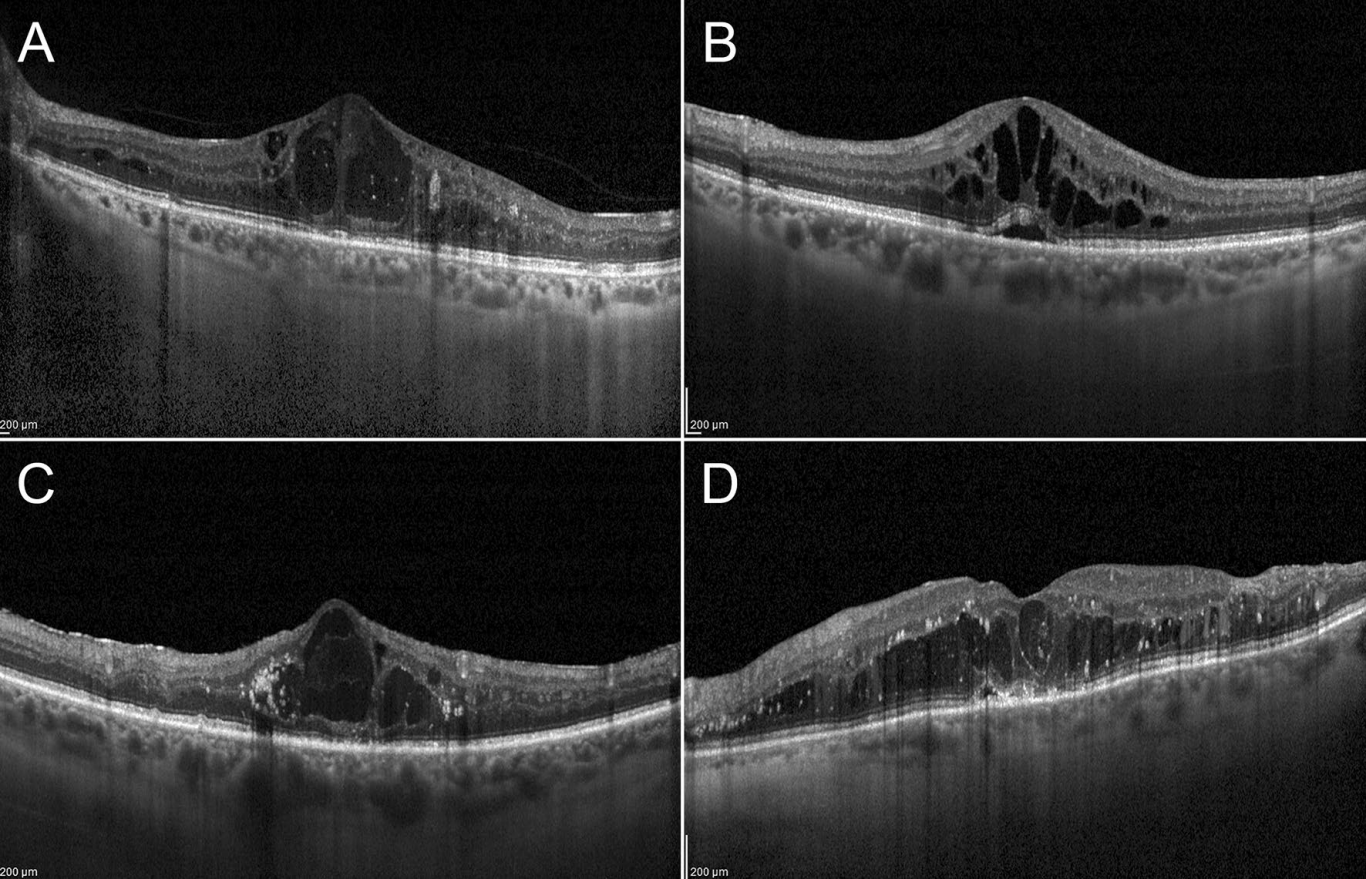
Diabetic macular edema categorization. Foveal eversion is classified as follows: Pattern 1a (**A**), showing a central columnar organization of the mixed reflectivity material; Pattern 1b (**B**), characterized by multiple thin central lines; and Pattern 2, showing no signs of central mixed reflectivity vertical material (**C**). A case of DME with no eversion of the foveal profile is shown in (**D**).

We analyzed eyes with and without foveal eversion, understood as the complete convex profile of the central fovea, separately. Taking cases displaying foveal eversion first, we considered three different patterns. Pattern 1 was defined as the presence of foveal eversion with mixed reflectivity, intraretinal, vertical material, developing in the context of the hyporeflective cystic space. We further subdivided Pattern 1, considering the columnar organization of the mixed reflectivity vertical signal (1a) or multiple fan-like lines (1b). Pattern 2 was defined as the presence of foveal eversion with only a hyporeflective intraretinal cystic signal. The DME categorization is shown in Fig. 1. Two independent graders (AA, AS) measured the horizontal thickness of the vertical mixed reflectivity material, assessing the thickness of all the mixed reflectivity lines included in the central 750 µm for both Pattern 1a and Pattern 1b. The grading of foveal eversion patterns was mandatorily done on a horizontal structural OCT b-scan image, centered to the fovea. DME eyes never showing foveal eversion over the entire study window were included in the “no foveal eversion” group.

DME eyes were treated with anti-VEGF (Ranibizumab 0.5 mg), dexamethasone (DEX) implant (Ozurdex^®^; Allergan inc.), or fluocinolone acetonide (FAc) 0.19 mg intravitreal drug-delivery system (ILUVIEN^®^; Alimera Sciences, Inc., Alpharetta, GA, USA), at the ophthalmologists’ discretion. Anti-VEGF injections and DEX implants were administered following a pro-re-nata regimen. The criterion employed to switch the treatment was the OCT-based evidence of poor response to the first molecule (arbitrarily considering DME reduction < 25%). FAc implant was administered according to the Italian guidelines, consisting of the following criteria: refractory DME, previously treated with at least one DEX implant, with no evidence of “cortico-responder”-related phenomena, in mandatory pseudophakic eyes. FAc-treated eyes might undergo further anti-VEGF treatment if displaying structural OCT evidence of poor response (in this case too, regarded as DME reduction < 25%).

To make all the eyes reliably comparable in terms of image quality and visual changes, being bound by the Italian criteria for FAc implant, we considered mandatory pseudophakia for eyes treated by anti-VEGF/DEX too. Likewise, we chose to exclude naïve DME eyes to further guarantee a reliable statistical comparison between anti-VEGF/DEX and FAc implant eyes.

The main outcome measure was the assessment of the relationship between presence/absence of foveal eversion, and the clinical course of DME. The secondary outcome was the assessment of the relationship between each foveal eversion pattern (1a, 1b and 2), and both the final outcome (DME resolving or recurrence) and the presence of persistent DME, understood as DME enduring throughout the 24-week examination^15^.

We considered the parameters measured at baseline and at the last follow-up. Two independent blinded graders performed all the measurements (AA, AS). Interclass correlation coefficient (ICC) was measured to assess the agreement between the two graders through a two-way random-effects model. For the statistical analyses we included the following variables: age, gender, systemic arterial hypertension, type and duration of diabetes mellitus (DM), glycate hemoglobin values (HbA1c), DR type (NPDR/PDR), previous history of vitrectomy, previous panretinal photocoagulation (PRP), previous nature and number of treatments (anti-VEGF, intravitreal corticosteroids), baseline features (BCVA and CMT), retinal and/or choroidal HF > 15, ERM, DRIL, ELM/EZ status (normal/interrupted/absent) and SRF. For the FAc-treated eyes, we also considered the presence of any additional anti-VEGF treatments (yes/no) and their number, administered at the ophthalmologists’ discretion.

All the statistical analyses were performed by means of the SPSS software package (SPSS, Illinois, USA). In our statistical models, we evaluated the normality distribution of each variable with frequency histograms and quantile–quantile plots. Descriptive statistics of continuous variables were reported as mean ± standard deviation, whereas frequency and proportions were described as categorical variables. Continuous variables were statistically evaluated using the two-tailed T-test. One-way ANOVA analysis assessed the differences between eyes displaying foveal eversion (considering all the foveal eversion patterns separately) and eyes without foveal eversion. The multiple testing required the introduction of Bonferroni correction in order to provide multiple comparisons. Tau-Kendall correlation analysis was adopted to assess the relationship between the various parameters. Statistical significance was set at p < 0.05.

## Results

Overall, 146 eyes of 146 patients affected by DME (80 males; mean age 67 ± 8 years) were recruited for the study. Eighty-four DME eyes (45 males; mean age 67 ± 6 years) were treated with anti-VEGF injections and DEX implants, whereas 62 eyes (35 males; mean age 68 ± 8 years) were treated with FAc implant.

The mean number of intravitreal injections before inclusion in the study was 12 ± 3. The first DME diagnosis was performed 2 ± 1 years before our baseline visit. DME eyes treated by anti-VEGF/DEX received a mean of 11 ± 4 injections over the entire follow-up. Sixty-nine out of 84 eyes (82%) underwent treatment, switching over the 2-year follow-up. Twenty-one out of 62 eyes (34%) in the FAc-treated group required additional anti-VEGF treatments (3 ± 1 injections). Clinical data are shown in Table 1.

**Table 1:** Clinical data in anti-VEGF/DEX and FAc-treated DME eyes.

Clinical data					
Parameter	Anti-VEGF/DEX group		FAc implant group		p value anti-VEGF/DEX vs FAc implant groups
	MEAN ± STD	p value	MEAN ± STD	p value	
Number of eyes	84		62		< 0.05
Age	67 ± 6		68 ± 8		> 0.05
Gender (M/F)	45/39	> 0.05	35/27	> 0.05	> 0.05
DM type (I/II) %	43%/57%	< 0.01	41%/59%	< 0.01	> 0.05
DR type (NPDR/PDR) %	59%/41%	> 0.05	40%/60%	> 0.05	< 0.05
Hb1Ac value %	7.2 ± 0.8		7.4 ± 0.8		> 0.05
Arterial hypertension %	67%		61%		> 0.05
Previous focal/grid laser %	27%		32%		> 0.05
Previous anti-VEGF %	95%		87%		> 0.05
Previous DEX %	81%		95%		> 0.05
Previous PRP %	52%		48%		> 0.05
Previous vitrectomy %	21%		16%		> 0.05
LogMAR BCVA baseline	0.56 ± 0.39	< 0.05	0.53 ± 0.38	< 0.05	> 0.05
LogMAR BCVA 2-year F/U	0.40 ± 0.31		0.42 ± 0.31		> 0.05
CMT baseline	564 ± 125	< 0.01	503 ± 131	< 0.01	> 0.05
CMT 2-year F/U	314 ± 120		327 ± 118		> 0.05
DME duration > 3 m %	90%		89%		> 0.05
DRIL baseline %	19%	< 0.05	15%	< 0.05	> 0.05
DRIL 2-year F/U %	38%		28%		> 0.05
EZ/ELM status baseline % (preserved/disrupted/absent)	29%/71%/0%	> 0.05	28%/72%/0%	> 0.05	> 0.05
EZ/ELM status 2-year F/U % (preserved/disrupted/absent)	31%/61%/8%		29%/61%/10%		> 0.05
SRF baseline %	26%	< 0.01	23%	< 0.01	> 0.05
SRF 2-year F/U %	0%		0%		> 0.05
HF > 15 baseline %	93%	> 0.05	94%	> 0.05	> 0.05
HF > 15 2-year F/U %	70%		77%		> 0.05
ERM baseline %	25%		23%		> 0.05
Foveal Eversion baseline %	54%	> 0.05	42%	> 0.05	> 0.05
Foveal Eversion 2-year F/U %	62%		50%		> 0.05
Additional anti-VEGF treatment %	N/A		34%		N/A
Persistent DME 2-year F/U	55%		50%		> 0.05

*DR* diabetic retinopathy; *DME* diabetic macular edema, *PRP* panretinal photocoagulation, *DEX* dexamethasone, *BCVA *best corrected visual acuity, *CMT* central macular thickness, *DRIL* disorganization of retinal inner layers, *EZ* ellipsoid zone, *ELM* external limiting membrane, *SRF* subretinal fluid, *HF* hyperreflective foci, *ERM* epiretinal membrane.

DME eyes showed statistically significant improvements of LogMAR BCVA and CMT over the 2-year follow-up (p < 0.05) (Table 1). No significant differences were detected looking at each administered drug (p > 0.05).

We found foveal eversion in 83 out of 146 eyes (57%). DME eyes with and without foveal eversion started with similar baseline BCVA values (p > 0.05) (Table 2). No significant differences among subgroups were found looking at DM Type, DR Type and previous treatments (all p > 0.05). No significant difference was detected in the number of intravitreal treatments administered in each subgroup (p > 0.05).

**Table 2:** Foveal eversion sub-analysis in DME eyes and clinical parameters.

	Foveal eversion			No foveal eversion
	Pattern 1a	Pattern 1b	Pattern 2	
N. of eyes baseline (%)	23 (32%)	16 (23%)	32 (45%)	75
N. of eyes last F/U (%)	16 (19%)	22 (27%)	45 (54%)	63
Age	64 ± 9	65 ± 8	69 ± 7	67 ± 8
LogMAR BCVA baseline	0.51 ± 0.33	0.52 ± 0.39	0.61 ± 0.33	0.50 ± 0.32
LogMAR BCVA last	0.36 ± 0.29	0.39 ± 0.28	0.52 ± 0.32	0.36 ± 0.30
Resolved DME	5 (31%)	1 (5%)	0 (0%)	14 (22%)
Recurrent DME	5 (31%)	7 (32%)	6 (13%)	39 (62%)
Persistent DME	6 (38%)	14 (64%)	39 (87%)	10 (16%)

p Values	Pattern 1a vs 1b	Pattern 1a vs 2	Pattern 1b vs 2	Foveal Eversion vs no Foveal Eversion
Age	> 0.05	> 0.05	> 0.05	> 0.05
LogMAR BCVA baseline	> 0.05	< 0.05	< 0.05	< 0.05
LogMAR BCVA last	> 0.05	< 0.05	< 0.05	< 0.05
Resolved DME	< 0.05	< 0.05	> 0.05	< 0.05
Recurrent DME	> 0.05	< 0.05	< 0.05	< 0.05
Persistent DME	> 0.05	> 0.05	> 0.05	> 0.05

DME eyes are divided accordingly with the presence and type of foveal eversion, independently from the treatments.

*BCVA *best corrected visual acuity, *DME* diabetic macular edema.

**Figure 2:**
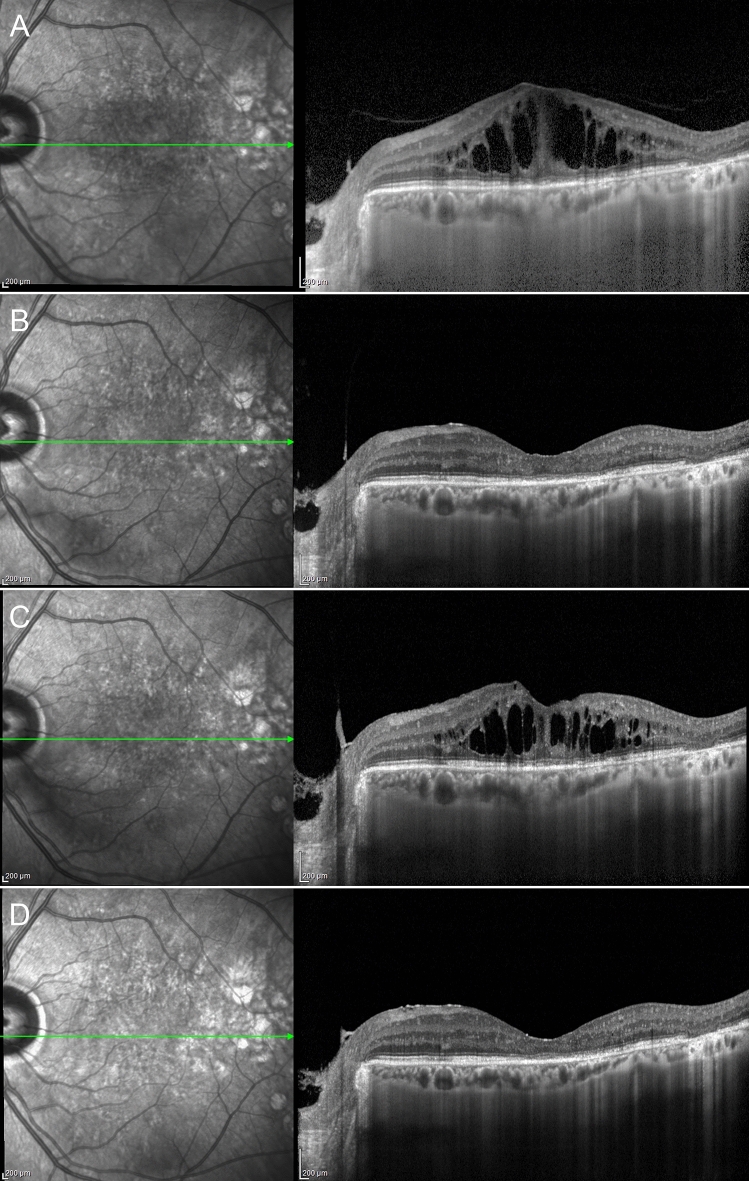
Foveal eversion Pattern 1a. The baseline condition is characterized by a DME with everted foveal profile and a central column of transretinal mixed reflectivity material (**A**). This case displays recurrent behavior, with complete regression of the intraretinal fluid (**B**) (6-month F/U) and further reappearance with preserved central columnar organization (**C**) (9-month F/U). At the last follow-up, DME has been completely reabsorbed and the ellipsoid zone is in good condition, despite a thinning of the outer nuclear layer (**D**).

**Figure 3:**
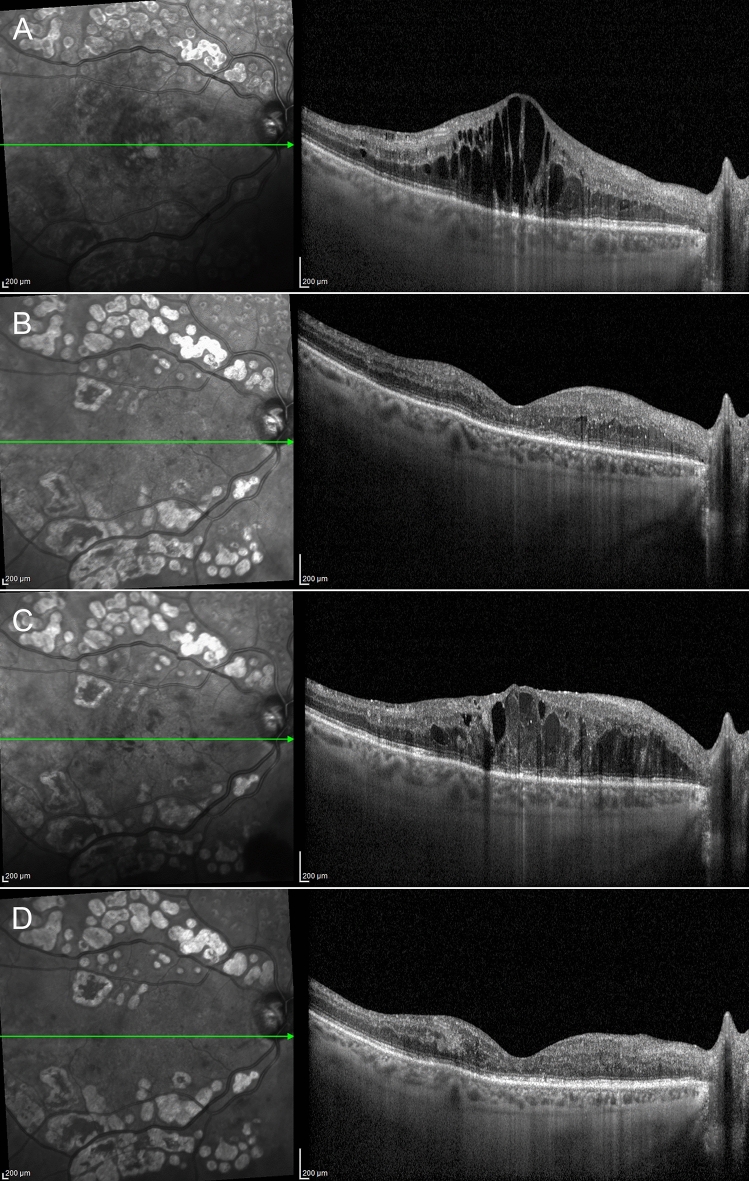
Foveal eversion Pattern 1b. Baseline examination shows a DME with foveal eversion and multiple thin transretinal mixed reflectivity central lines (**A**). Fluid reabsorption is registered after treatment (**B**) (6-month F/U) and DME shows recurrence during the follow-up, with disorganization of the retinal layers in the foveal region (**C**) (9-month F/U). At the last follow-up, DME has regressed, and evident DRIL, attenuated ellipsoid zone and thinning of the outer nuclear layer are detected (**D**).

**Figure 4:**
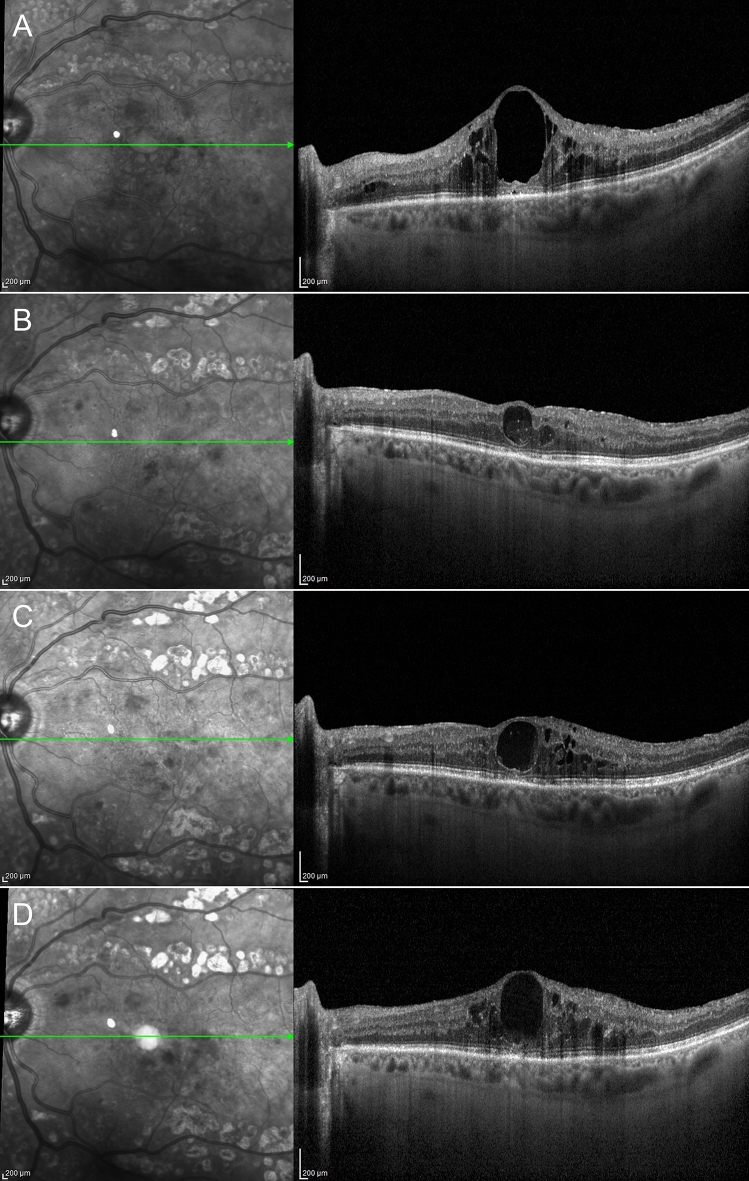
Foveal eversion Pattern 2. Baseline image shows foveal eversion with no signs of vertical transretinal foveal material (**A**). Although responsive to treatment, this DME shows persistent clinical behavior (**B**,**C**) over the follow-up (6-month and 9-month F/U, respectively). The last examination shows a disrupted ellipsoid zone at the level of the fovea (**D**).

**Figure 5:**
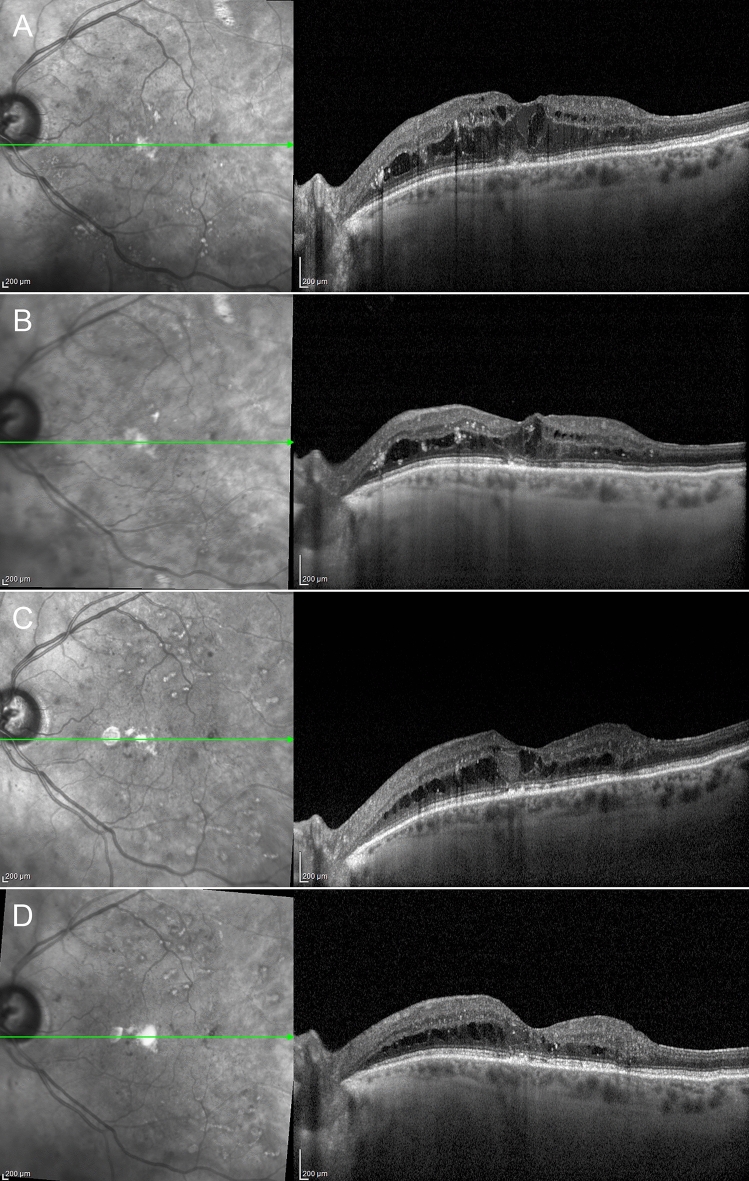
A case of diabetic macular edema with no foveal eversion. Baseline examination shows a DME with no everted foveal profile (**A**). The clinical evolution of this case is characterized by a persistence of the intraretinal fluid (**B**,**C**) (6-month and 9-month F/U, respectively), although the foveal region appears partially spared at the last follow-up (**D**).

DME eyes showing foveal eversion were categorized as follows: Pattern 1a (23, 32%) (Fig. 2); Pattern 1b (16, 23%) (Fig. 3) and Pattern 2 (32, 45%) (Fig. 4) at baseline, turning into Pattern 1a (16, 19%); Pattern 1b (22; 27%) and Pattern 2 (45, 54%) at the end of the follow-up. One case of DME without foveal eversion is shown in Fig. 5. Age and disease duration were similar among groups (all p > 0.05). Pattern 2 showed the worst baseline BCVA (p < 0.05), whereas Pattern 1a, Pattern 1b and eyes without foveal eversion started with similar BCVA values (p > 0.05). All the subgroups, except Pattern 2, underwent statistically significant BCVA improvement at the end of the follow-up (p < 0.01). The entire cohort of eyes also showed significant CMT recovery (p < 0.01). The distribution of all the other parameters are extensively reported in Tables 2 and 3. It is worth noting that the disappearance of the EZ/ELM was significantly higher in Pattern 2 (p < 0.01). SRF had disappeared in all eyes by the end of the follow-up. The percentage of eyes showing resolved DME was significantly higher in eyes without foveal eversion (p < 0.01). Most of the eyes included in Pattern 1a and foveal eversion-free subgroups were characterized by recurrent DME (p < 0.01), whereas most of the eyes included in Pattern 1b and Pattern 2 featured persistent DME (64% and 87% of cases, respectively; p < 0.01).

**Table 3:** Foveal eversion sub-analysis in DME eyes and imaging parameters.

	Foveal Eversion			No Foveal Eversion
	Pattern 1a	Pattern 1b	Pattern 2	
CMT baseline	498 ± 91	548 ± 115	582 ± 122	520 ± 112
CMT last	276 ± 58	380 ± 110	416 ± 112	271 ± 67
DRIL baseline (%)	3 (13%)	5 (31%)	11 (34%)	6 (8%)
DRIL last (%)	6 (38%)	12 (55%)	23 (51%)	8 (13%)
EZ/ELM status baseline % (preserved/disrupted/absent)	3 (13%)/20 (87%)/0 (0%)	4 (25%)/12 (75%)/0 (0%)	11 (34%)/19 (59%)/2 (6%)	30 (40%)/45 (60%)/0 (0%)
EZ/ELM status last % (preserved/disrupted/absent)	6 (38%)/9 (56%)/1 (6%)	8 (36%)/11 (50%)/3 (14%)	16 (36%)/29 (42%)/10 (22%)	22 (35%)/39 (62%)/2 (3%)
SRF baseline %	5 (21%)	10 (61%)	16 (50%)	17 (23%)
SRF last %	0 (0%)	0 (0%)	4 (9%)	0 (0%)
HF > 15 baseline %	20 (87%)	16 (100%)	32 (100%)	39 (52%)
HF > 15 last %	6 (37%)	13 (59%)	36 (80%)	47 (75%)
Vertical mixed reflectivity material thickness baseline	174 ± 37	120 ± 88	0 ± 0	0 ± 0
Vertical mixed reflectivity material thickness last	152 ± 52	81 ± 58	0 ± 0	0 ± 0
ERM baseline %	7 (30%)	9 (56%)	5 (21%)	32 (43%)

p values	Pattern 1a vs 1b	Pattern 1a vs 2	Pattern 1b vs 2	Foveal Eversion vs No Foveal Eversion
CMT baseline	> 0.05	< 0.05	> 0.05	< 0.05
CMT last	< 0.05	< 0.05	> 0.05	< 0.05
DRIL baseline (%)	< 0.05	< 0.05	> 0.05	< 0.05
DRIL last (%)	< 0.05	< 0.05	> 0.05	< 0.05
EZ/ELM status baseline % (preserved/disrupted/absent)	> 0.05	> 0.05	> 0.05	< 0.05
EZ/ELM status last % (preserved/disrupted/absent)	< 0.05	< 0.05	< 0.05	< 0.05
SRF baseline %	< 0.05	< 0.05	> 0.05	< 0.05
SRF last %	> 0.05	< 0.05	< 0.05	< 0.05
HF > 15 baseline %	> 0.05	> 0.05	> 0.05	< 0.05
HF > 15 last %	> 0.05	> 0.05	< 0.05	< 0.05
Vertical mixed reflectivity material thickness baseline	> 0.05	N/A	N/A	N/A
Vertical mixed reflectivity material thickness last	> 0.05	N/A	N/A	N/A
ERM baseline %	> 0.05	> 0.05	< 0.05	> 0.05

DME eyes are divided accordingly with the presence and type of foveal eversion, independently from the treatments.

*DME* diabetic macular edema, *CMT* central macular thickness, *DRIL* disorganization of retinal inner layers, *SRF* subretinal fluid, *HF* hyperreflective foci, *EZ* ellipsoid zone, *ELM* external limiting membrane.

In the three patterns of foveal eversion correlated with Hb1Ac level, we found that the higher the Hb1Ac value, the higher the foveal eversion pattern (Tau–Kendall coeff. 0.455; p < 0.001). Foveal eversion patterns showed negative correlation with the thickness of the mixed reflectivity vertical materials, Pattern 1a being the thickest (Tau–Kendall coeff. 0.732; p < 0.001). Furthermore, we found that the thinner the mixed reflectivity material, the worse the DME prognosis (looking at DME resolution, recurrence, or persistence) (Tau–Kendall coeff. 0.644; p < 0.001). Moreover, foveal eversion patterns significantly correlated with EZ/ELM status (Tau–Kendall coeff. 0.402; p < 0.001). As expected, the latter significantly correlated with BCVA values (Tau–Kendall coeff. 0.536; p < 0.001). The presence of SRF showed a moderate correlation with DME persistence (Tau–Kendall coeff. 0.398; p < 0.01); however, it had no significant influence on foveal eversion patterns (p > 0.05). Similarly, we found no significant correlations between foveal eversion patterns and all the other systemic and structural OCT parameters investigated (all p > 0.05).

There was high inter-grader agreement in the foveal eversion pattern classification (ICC 0.95; p < 0.01). The overall ICC regarding the other parameters analyzed was 0.94 (range 0.89–0.96; p < 0.01).

**Figure 6:**
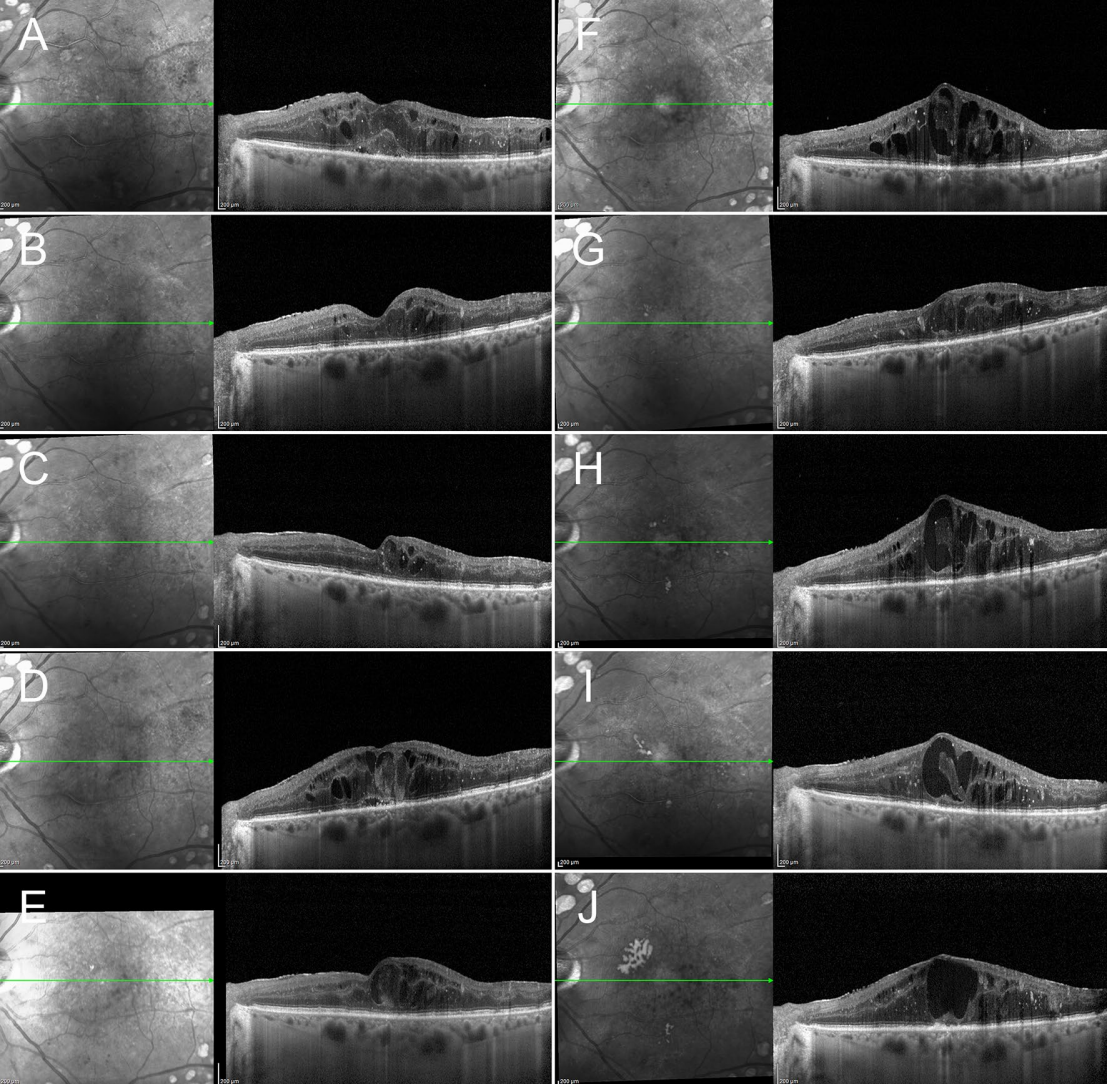
The possible pathogenesis of foveal eversion in diabetic macular edema. The starting condition is a DME (**A**) responding to intravitreal treatments, with recurrence/persistence of clinical behavior (**B**,**C**). The persistence of the intraretinal fluid leads to a progressive disorganization of both inner and outer retinal layers (**D**,**E**). Although the amount of DME in the recurrence (**D**) is similar to the baseline condition (**A**), its recurrence occurs in the context of a more disorganized retina, showing increased intraretinal hyperreflective foci and vertical linear mixed reflectivity signals in the context of the edema, which might be interpreted as a gliotic reaction of the foveal Muller cells (**D**). The subsequent partial regression of the edema (**E**) is characterized by still higher intraretinal disorganization than the previous similar partial regression (**B**). Foveal eversion then appears (**F**), in this case categorizable as Pattern 1b (multiple transretinal thin lines). The onset of foveal eversion occurs together with a focal central disruption of the ellipsoid zone, highlighted by choroidal hyper-transmission detected on structural OCT (**F**,**G**). In later stages, progressive degenerative phenomena occur within the foveal region (**H**,**I**), leading the condition to evolve into a Pattern 2 foveal eversion (no signs of transretinal vertical material) and further disruption of the ellipsoid zone (**J**).

Consecutive follow-up examinations, starting from a DME without foveal eversion, evolving into a DME with foveal eversion, prompted us to put forward the possible pathogenic mechanism shown in Fig. 6.

## Discussion

Foveal eversion is a relatively new structural OCT biomarker and was previously related to worse DME outcomes with higher prevalence of persistent DME^15,16^. In the present study, we carried out a more thorough investigation of this parameter, in an attempt to further categorize DME eyes according to their morphological features. We detected three different patterns of foveal eversion. Pattern 1 was characterized by the presence of vertical, transretinal, mixed reflectivity material, associated with a cystoid edema, organized as a single thicker columnar unit (Pattern 1a) or in multiple thinner lines (Pattern 1b). Pattern 2 was characterized by the absence of this mixed reflectivity material, with only a hyporeflective cystoid intraretinal signal. Although the overall prevalence of persistent DME reported in our study (almost 50% of the cases) was in line with previous investigations^15,17^, the presence of foveal eversion was significantly associated with persistent DME, reaching the highest prevalence in Pattern 1b (64% of the cases) and in Pattern 2 (87% of the cases). In contrast, Pattern 1a and eyes without foveal eversion were mainly characterized by the recurrence of DME.

In our previous investigation, we speculated about the eversion of the foveal profile’s possible association with Muller cell impairment^16^. The theoretical basis for this hypothesis lies in the fact that Muller cells are a key structural and homeostatic component of the human retina^18,19^, being directly involved in DME pathogenesis and responsible for the morpho-functional integrity of the foveal region^18,20–22^. In the current study, we hypothesize that Muller cells might be variably impaired, as clinically expressed by three different patterns of foveal eversion. In particular, the absence of the vertical transretinal mixed reflectivity material characterizing Pattern 2, interpreted as the total disruption of foveal Muller cells, was significantly associated with the worst morpho-functional outcome. Conversely, the presence of this vertical signal, detected either as thick column (Pattern 1a) or thinner lines (Pattern 1b) might be interpreted as the structural OCT sign of Muller cells partial sparing. The overall thickness of the vertical transretinal signal was significantly associated with better morpho-functional outcome. The impairment of the foveal region occurred independently of the administered drug, the duration of the disease and the number of injections. This might mean that other factors in the multifaceted DME pathogenesis might be responsible for the progressive loss of foveal integrity^23^. The eversion of the fovea might be the consequence of gliotic damage involving the Muller cells^22^, together with a different inflammatory profile typifying this category of DME eyes^24^. The most intriguing aspect of the present investigation is that different patterns of foveal eversion may allow to identify the prognostic information about the patient's morpho-functional recovery after treatments. In this perspective, foveal eversion might represent a feasible OCT-based retinal biomarker, potentially offering the basis for an efficient and personalized DME management.

However, we must acknowledge that further, multidisciplinary studies are needed in order to draw more definite conclusions about the role of foveal eversion in DME pathogenesis.

We are aware that our study labors under several limitations, related firstly to the relatively low number of DME eyes and the strict follow-up. Furthermore, our investigation was conducted exclusively on eyes with a history of DME; no dedicated information about naïve DME is available. We made this choice so we could include eyes treated by FAc implant, and thus try to provide a comprehensive scenario of foveal eversion’s role in DME treated with all the current therapeutic strategies. Future prospective studies should focus on assessing foveal eversion’s features, as well as its role in naïve DME eyes. Moreover, since most of the eyes underwent treatment switching, we were unable to provide data about the part played by foveal eversion in the decisions regarding treatment switching. Furthermore, future interventional trials are warranted to evaluate the effect of customized treatments, based on foveal eversion detection, on the clinical outcome of DME. Lastly, we proposed a possible pathogenic evolution of DME from a condition in which foveal eversion is absent to one in which it is present. However, since our considerations were mainly based on structural OCT follow-ups, histological validation is required to draw more definite conclusions about the progressive development of foveal eversion in DME.

In conclusion, we confirmed the role of foveal eversion as a feasible and useful biomarker requiring validation in a DME clinical setting. The everted fovea is likely related to the impairment of the Muller cells, with consequent loss of foveal homeostasis and worse DME outcome. In the interest of designing ever more optimized and personalized treatment strategies, foveal eversion may be considered a useful biomarker in DME eyes. Further studies are needed to provide a more thorough assessment of the significance of foveal eversion in the pathogenesis of DME.
